# Improved iterative reconstruction method for Compton imaging using median filter

**DOI:** 10.1371/journal.pone.0229366

**Published:** 2020-03-06

**Authors:** Makoto Sakai, Raj Kumar Parajuli, Yoshiki Kubota, Nobuteru Kubo, Mikiko Kikuchi, Kazuo Arakawa, Takashi Nakano

**Affiliations:** 1 Gunma University Heavy Ion Medical Center, Graduate School of Medicine, Gunma University, Showa-machi, Maebashi, Gunma, Japan; 2 Department of Molecular Imaging and Theranostics, National Institutes for Quantum and Radiological Science and Technology, Anagawa, Inage, Chiba, Japan; 3 Department of Radiation Oncology, Gunma University Graduate School of Medicine, Showa-machi, Maebashi, Gunma, Japan; Chongqing University, CHINA

## Abstract

A Compton camera is a device for imaging a radio-source distribution without using a mechanical collimator. Ordered-subset expectation-maximization (OS-EM) is widely used to reconstruct Compton images. However, the OS-EM algorithm tends to over-concentrate and amplify noise in the reconstructed image. It is, thus, necessary to optimize the number of iterations to develop high-quality images, but this has not yet been achieved. In this paper, we apply a median filter to an OS-EM algorithm and introduce a median root prior expectation-maximization (MRP-EM) algorithm to overcome this problem. In MRP-EM, the median filter is used to update the image in each iteration. We evaluated the quality of images reconstructed by our proposed method and compared them with those reconstructed by conventional algorithms using mathematical phantoms. The spatial resolution was estimated using the images of two point sources. Reproducibility was evaluated on an ellipsoidal phantom by calculating the residual sum of squares, zero-mean normalized cross-correlation, and mutual information. In addition, we evaluated the semi-quantitative performance and uniformity on the ellipsoidal phantom. MRP-EM reduces the generated noise and is robust with respect to the number of iterations. An evaluation of the reconstructed image quality using some statistical indices shows that our proposed method delivers better results than conventional techniques.

## 1. Introduction

A Compton camera is a device for imaging a radio-source distribution without using a mechanical collimator. An elementary Compton camera contains two types of position-sensitive detectors. A Compton event consists of Compton scattering in the first detector (scatterer) and absorption in the second detector (absorber). The scatterer and the absorber record the interaction positions and deposited energies for each event. When an electron is assumed to be free and at rest, the scattering angle *θ* in the scatterer can be calculated from

$$cos\theta =1-\frac{m_{e}c^{2}E_{1}}{E_{2}(E_{1}+E_{2})}$$ (1)

where *m*_e_c^2^ is the mass-energy of an electron, *E*_1_ is the energy of the recoiled electron in the scatterer, and *E*_2_ is the energy deposited in the absorber. The direction of the incident gamma ray is restricted to an area called a “Compton cone”.

An application of this type of camera in nuclear medicine was first proposed by Todd et al. [1]. As no mechanical collimator is required in a Compton camera, simultaneous imaging of multiple radionuclides is possible over a wide field of view, with high efficiency, and across a wide energy range (from several tens of keV to a few MeV). Thus, Compton cameras are a promising mode of medical imaging [2].

Compton cameras have been developed for use in the field of γ-ray astronomy [3, 4]. We have been developing a semiconductor-based Compton camera for a nuclear medical imaging system that was originally developed by the Japan Aerospace eXploration Agency (JAXA) [5–7]. Our Compton camera used a silicon (Si) semiconductor detector with low noise, allowing low-energy gamma emitters to be imaged with high accuracy [8].

In recent years, the imaging ability of Compton cameras has rapidly improved and realistic experiments have been performed, even though the development of medical Compton cameras has a long history. Several reconstruction methods have been proposed for Compton imaging. Unfortunately, the current research on reconstruction methods for Compton cameras has, to date, not made significant progress. Among them, maximum-likelihood expectation-maximization (ML-EM) and ordered-subset expectation-maximization (OS-EM) are commonly used imaging algorithms. However, ML-EM and OS-EM are prone to amplifying the noise through excessive iterations, leading to difficulties in evaluating the imaging ability because the optimal number of iterations is not known in advance. In recent studies, the number of iterations tends to be arbitrarily assigned, considering noise and calculation time. This makes it difficult to compare the image qualities in a fair and scientific manner. Thus, we developed a reconstruction algorithm, which can reduce the emphasis on noise. Iterative post-smoothed methods reduce the noise, but cannot solve the problem of over-convergence fundamentally. Therefore, in clinical use, it would be beneficial to use a priori knowledge that could supress the noisy parameters in image reconstruction. This prior knowledge is somehow inserted in the reconstruction algorithms for single photon emission computed tomography (SPECT) and positron emission tomography (PET) images [9–12], but not utilized in Compton imaging. Median filters can efficiently reduce noise levels. Therefore, in this study, we developed a noise-reducing imaging algorithm by adding a median filter to the OS-EM algorithm (median root prior EM, MRP-EM) for Compton imaging. The quality of the images reconstructed by implementing the proposed advanced method was tested using computer simulations. Spatial resolution, several image quality indices, the semi-quantitative ability, and uniformity of MRP-EM were evaluated using an ellipsoid phantom to ensure that the proposed method can be used effectively in nuclear medicine. MRP-EM was compared with simple backprojection (BP), OS-EM, and the stochastic origin ensemble (SOE) method [13–15] as well as with the analytical method developed by Tomitani and Hirasawa [16–18].

## 2. Methods

### 2.1 Monte Carlo simulations

**Fig 1:**
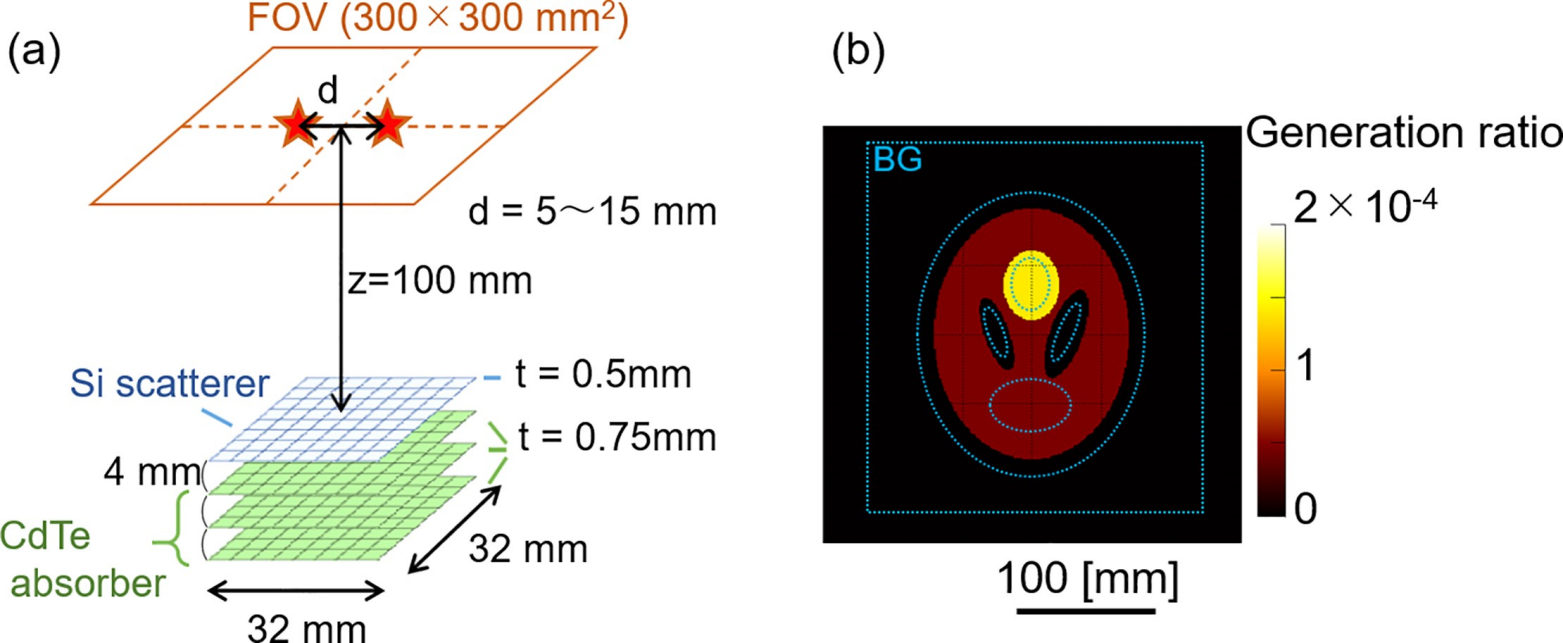
Imaging setup (a) and source distribution of ellipsoid phantom (b). The blue dotted line in (b) represents the region of interest for the semi-quantitative and uniformity tests.

To generate data for testing our algorithm, Geant4-based Monte Carlo simulations were conducted [19]. The simulation codes used to emulate our existing Compton camera have already been verified in previous studies [6, 7, 20, 21]. Fig 1(A) shows the geometry of the simulation model. The Compton camera consists of a single-layer Si detector and a three-layer cadmium telluride (CdTe) detector. The active area of each detector is 32 × 32 mm^2^ divided into 128 strips on each side. The Si and CdTe detectors were 500 μm and 750 μm thick, respectively, with 4-mm spaces between them. Simulations were performed considering the realistic conditions that usually occur during Compton camera measurements, where the data are affected by segmented position determination, limited resolution, and Doppler broadening. The typical energy resolution (full width at half-maximum) was 3.8 keV at 81.0 keV and the angular resolution measurement (ARM) was 4.9° at 511 keV [7]. Further specifications have been described by Odaka et al. [22].

In this study, only successive events, involving the Si scatterer and one of the CdTe absorbers, were considered in list mode for image reconstruction. A photo-peak energy window of 20 keV (501–521 keV) was applied to sum the measured energies in the scatterer and absorber.

We performed simulations on two types of mathematical phantoms. The first one contained two point sources of 511 keV gamma rays placed in line at y = 0, with a separation distance ranging from 5–15 mm, shifted by 1 mm each. The reconstructed images obtained through this simulation were used to evaluate the spatial resolution. The other phantom was an ellipsoidal phantom, similar to a Shepp-phantom [23]. As shown in Fig 1(B), this consisted of a hot-spot (HS) (yellow region) and two cold spots (CS1, CS2) (black region) in a large ellipsoid region (LE) (dark red region). The activity concentration of HS was 3.5 times that in LE, and the activity concentration of the remaining parts, including the two cold spots, was zero. All sources were placed in a plane parallel to the plane of the detectors and were 100 mm apart from the plane of the first detector (Si scatterer).

### 2.2 Imaging algorithms

#### 2.2.1 General parameters

Images were reconstructed on the plane parallel to that of the detectors at a distance of 100 mm. The field-of-view (FOV) was 300 × 300 mm^2^ with a 1 × 1 mm^2^ pixel size. The reconstructed images were normalized by the sum of pixel values to transform the probability distribution so as to compare with the source distribution. Calculations were performed using a 2.7 GHz 12-core Intel Xeon E5 processor (using only one core).

#### 2.2.2 Simple backprojection

In Compton imaging, a Compton cone is reconstructed from the vector joining two interaction points and the scattering angle (calculated from the Compton kinematics of (1)). In general, the quadric curve described by the interaction between the Compton cone and imaging plane suggests the origin of the gamma ray. Because of the finite angular resolution of the Compton camera, there are some uncertainties regarding the original point. Therefore, we adopt a Voigt function to consider the uncertainty. The pixel value *λ*_*j*_ at the *j*th pixel can then be expressed as:

$$\lambda_{i,j}={\left|\vec{D}\right|}^{-2}V_{\left(\theta_{i};\sigma ,\gamma \right)}$$ (2)

$$\lambda_{j}=\sum_{i}\lambda_{i,j}$$ (3)

where i expresses the number of the event, $\vec{D}$ represents the vector from the apex of the Compton cone to the reference point, *V*(*θ*_*i*_;*σ*,*γ*) represents the Voigt profile, *σ* and *γ* are the parameters of ARM determined by a point source imaging examination, ${\left|\vec{D}\right|}^{-2}$ corrects the distance effect in near-field imaging, and *θ*_*i*_ is the minimum angular difference between $\vec{D}$ and the vector on the surface of the Compton cone of the *i*th event [7, 24].

#### 2.2.3 Ordered-subset expectation-maximization

The ML-EM algorithm is widely used for image reconstruction. Although OS-EM works in a similar way to ML-EM, the algorithm is optimized by performing an update after each subset of the total amount of data has been processed [25, 26]. For *L* subsets, these steps are calculated as follows:

$$\lambda_{j}^{\left(k,l+1\right)}=\frac{\lambda_{j}^{\left(k,l\right)}}{S_{j}}\sum_{i\in S_{l}}\frac{t_{ij}}{\sum_{m}t_{im}V_{m}^{\left(k,l\right)}}$$ (4)

$$\lambda_{j}^{\left(k+1,0\right)}=\lambda_{j}^{\left(k,L\right)}$$ (5)

where $\lambda_{j}^{\left(k,0\right)}$ is the pixel value in the image of the *k*th iteration, $\lambda_{j}^{\left(k,l\right)}$ is the updated value of the *k*th image using l subsets, *S*_*j*_ is the detection efficiency vector, and *t*_*ij*_ is the transition probability of the *i*th event at the reference point. *t*_*ij*_ is calculated in the same manner as in Eq (2). The detection efficiency vector ***Sj*** was calculated analytically, considering geometrical and physical conditions [7]. In this study, the BP image was considered as the initial image and the number of subsets was set to four on considering the number of Compton events and improvement of calculation time.

#### 2.2.4 Median root prior expectation-maximization

MRP is based on the one-step-late (OSL) algorithm [27]. OSL modifies OS-EM by incorporating prior information. MRP assumes that the most probable value of the pixel is close to the local median. In MRP-EM, the pixel value is calculated by the following equation:

$$\lambda_{j}^{\left(k,l+1\right)}=\frac{\lambda_{j}^{\left(k,l\right)}}{S_{j}(1+\beta \frac{\lambda_{j}^{\left(k,l\right)}-med(\lambda j)}{med(\lambda j)})}\sum_{i\in S_{l}}\frac{t_{ij}}{\sum_{m}t_{im}\lambda_{m}^{\left(k,l\right)}}$$ (6))

where β is a hyperparameter that influences the degree of smoothness of the estimated images, and *med*(*λj*) is the median of the image pixels over a neighbourhood around the *j*th pixel. In this study, β was set to 1 to accent the effect of median filter, and the size of the median mask was set to 7 × 7 (S1 Fig).

#### 2.2.5 Stochastic Origin Ensemble method

The SOE approach to Compton imaging based on Markov chains was developed by Andreyev [14, 15]. It does not require forward and backward projection operations. To make an initial image, the possible origin-points of all measured events are selected randomly on the corresponding conical surface in the imaging area. A new location of the *i*th event is then randomly selected from the possible origin positions. Finally, the pixel values (event density in the neighbourhood) *λ*_*i*,*k*_ and *λ’*_*i*,*k*_ of the former and the new candidate points in the *k*th image are compared, and the acceptance probability *A* is calculated as:

$$A=min\left(1,\frac{\lambda_{i,k}+1}{{\lambda \prime }_{i,k}}\right)$$ (7)

According to *A*, the presumed origin-point is changed to the new point or remains at the current location. After all values of *A* have been estimated for all events, the *k*+1th image is reconstructed. As the number of iterations increases, the SOE algorithm produces high-frequency noise. A Gaussian filter can be applied to the reconstructed images to suppress this noise.

#### 2.2.6 Analytical method

Tomitani and Hirasawa developed an analytical method using spherical harmonics. Their algorithm was later extended to compensate for angular uncertainties [16–18]. They computed

$$\lambda_{j}=\int_{cos\omega 2}^{cos\omega 1}d\;cos\omega \;\int_{S}\;d\vec{t}\;k^{-1}(\vec{t},\vec{p};\omega )g(\vec{t};\omega )$$ (8)

where *ω*_*1*_ and *ω*_*2*_ are the minimum and maximum scattering angles, respectively, in the reconstruction, *S* is a unit sphere centred at the scattered point, $\vec{t}$ denotes the direction of the unit vector on the projection, $g(\vec{t};\omega )$ is the projection data, and $k^{-1}(\vec{t},\vec{p};\omega )$ represents the cone transform, which is described as:

$$k^{-1}(\vec{t},\vec{p};\omega )=\sum_{n=0}^{\infty }\frac{2n+1}{4\pi }\frac{1}{H_{n}}P_{n}(cos\omega )P_{n}(\left(\vec{t},\vec{p}\right))$$ (9)

$$H_{n}=\int_{\omega 2}^{\omega 1}\sigma (cos\omega )P_{n}{\left(cos\omega \right)}^{2}dcos\omega$$ (10)

$$\sigma (cos\;\omega )=\frac{1+{cos}^{2}\omega }{2{\left\{1+(E_{1}+E_{2})(1-cos\omega )/m_{e}\right\}}^{2}}\left[1+\frac{{\left((E_{1}+E_{2})(1-cos\omega )/m_{e}\right)}^{2}}{\left(1+{cos}^{2}\omega )\{1+(E_{1}+E_{2})(1-cos\omega )/m_{e}\right\}}\right]$$ (11)

where P_n_ is the Legendre polynomial of order n. In this study, ω_1_ and ω_2_ were set to 5° and 90°, respectively.

### 2.3 Evaluation of the reconstruction quality

**Fig 2:**
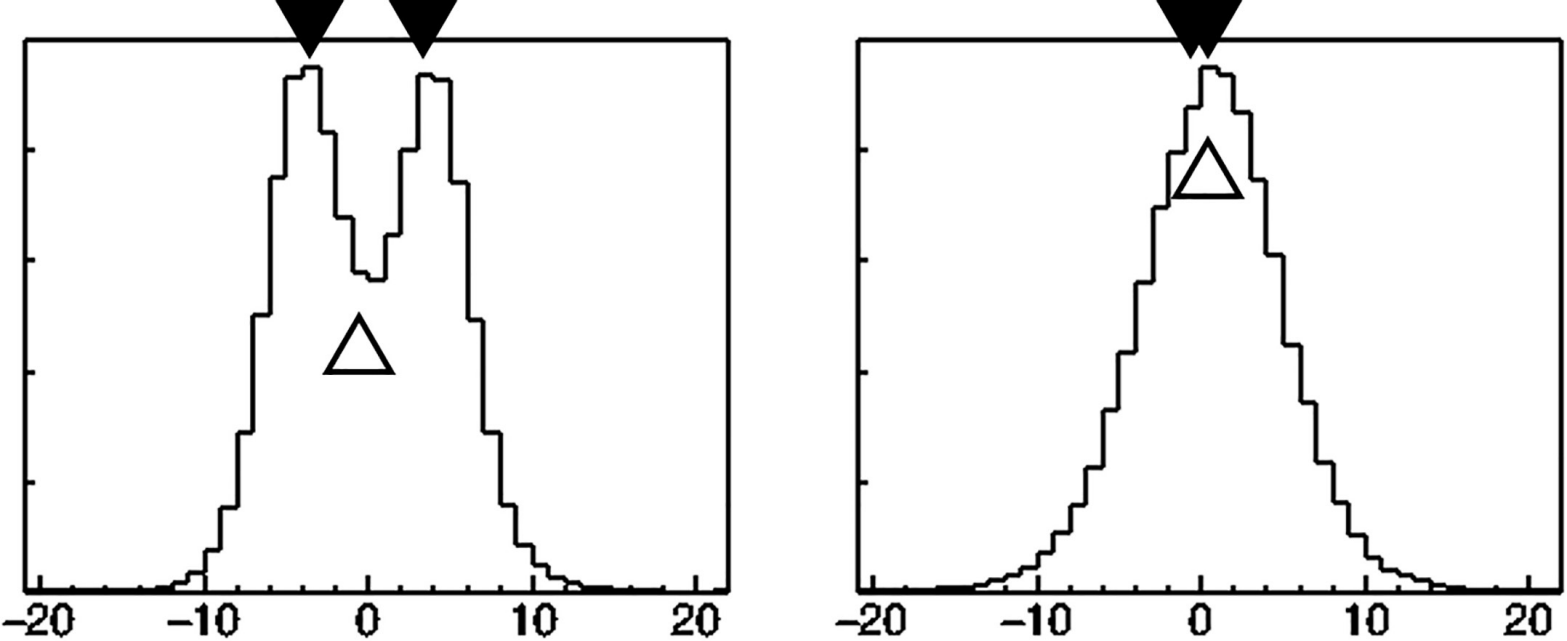
Examples of the line profile for spatial resolution analysis. The two point sources are said to be distinct when the maximum values in both x<0 and x>0 are larger than the value of x = 0 in the profile of y = 0 (the origin was defined as the centre of the images). (A): distinguishable, (B): indistinguishable.

To validate the spatial resolution, two 511 keV gamma-ray point sources were measured. They were located in a plane 100 mm from the first layer of the Si detector. The two point sources were placed in line at y = 0, with a distances of 5–15 mm apart. Using a line profile through the two point source positions in the reconstructed image for each method, the spatial resolution was defined as the minimum distance at which two peaks were visible. The two point sources are said to be distinct, when the maximum values in both x<0 and x>0 are larger than the value of x = 0 in the profile of y = 0 (the origin was defined as the centre of the images) (Fig 2).

For the imaging of the ellipsoidal phantom, the degree of coincidence with the original source distribution was evaluated using the following three indices: residual sum of squares (RSS), zero-mean normalized cross-correlation (ZNCC), and mutual information (MI). These indices were calculated as follows:

$$RSS=\sum {\left(V_{T}-V_{R}\right)}^{2}$$ (12)

$$ZNCC=\frac{\sum \left(V_{T}-\bar{V_{T}})(V_{R}-\bar{V_{R}}\right)}{\sqrt{\sum {\left(V_{T}-\bar{V_{T}}\right)}^{2}\sum {\left(V_{R}-\bar{V_{R}}\right)}^{2}}}$$ (13)

$$MI=\sum p_{TR}(t,r)∙log\frac{p_{TR}(t,r)}{p_{T}(t)∙p_{R}(r)}$$ (14)

where *V*_*T*_ and *V*_*R*_ are the pixel values of the true source distribution and the reconstructed image, respectively, $\bar{V_{T}}$ and $\bar{V_{R}}$ are the average pixel values, *p*_*T*_ and *p*_*R*_ are the marginal probability distributions of the pixel values, and *p*_*TR*_(*t*,*r*) is the joint probability distribution. In this study, MI was calculated for 256 gradation levels of the images. Good performance by an algorithm would result in a small RSS, ZNCC close to one, and high MI.

In addition, we evaluated the semi-quantity and uniformity performance. The averages and coefficient of variation (CV = Standard Deviation / Average) in the region of interest (ROI) (blue broken line in Fig 1(B)) were calculated.

## 3. Results

### 3.1 Point source

**Fig 3:**
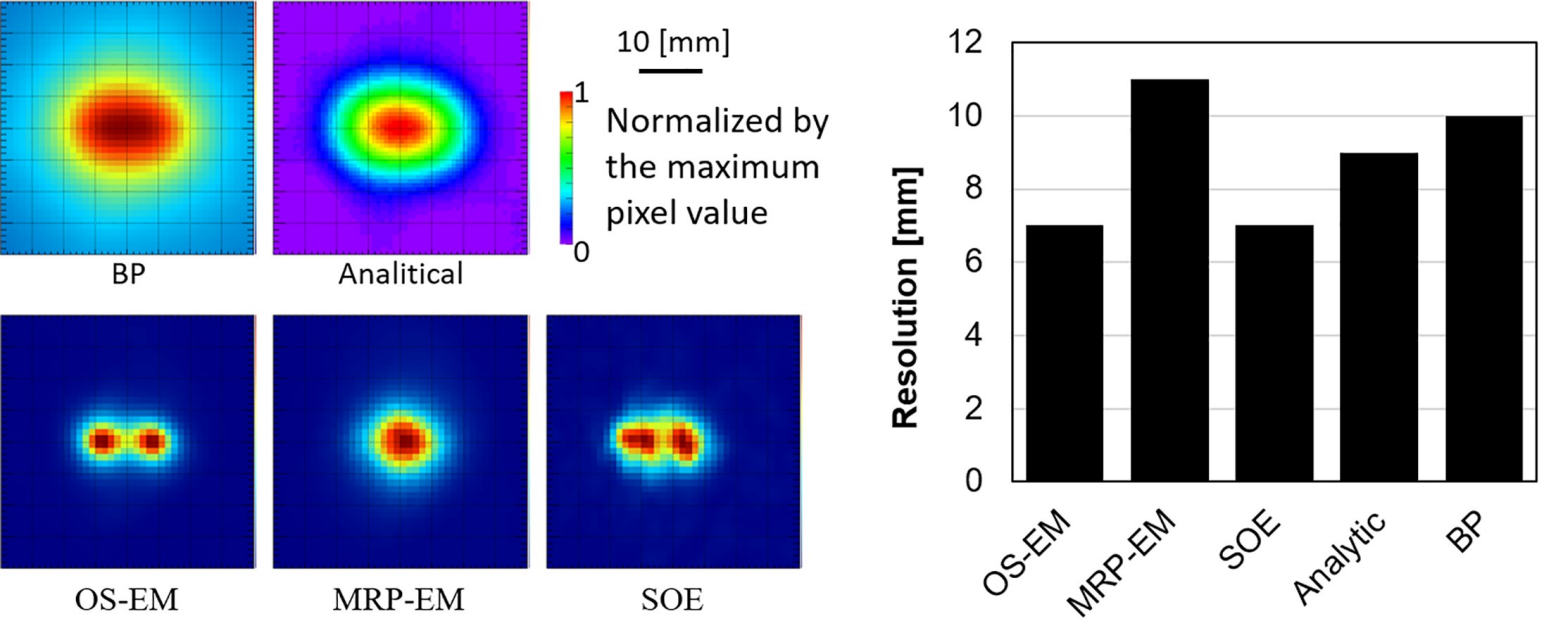
Images of two point sources with 8 mm distance (A)—(E) and spatial resolutions of the images reconstructed by the various algorithms (F).

In the Monte Carlo simulation, about 16000 Compton events were considered. The spatial resolutions determined from the two point sources are shown in Fig 3. Among OS-EM, MRP-EM, SOE, and Analytic and BP algorithms, OS-EM and SOE algorithms were found to have the best spatial resolutions.

### 3.2 Ellipsoidal phantom study

**Fig 4:**
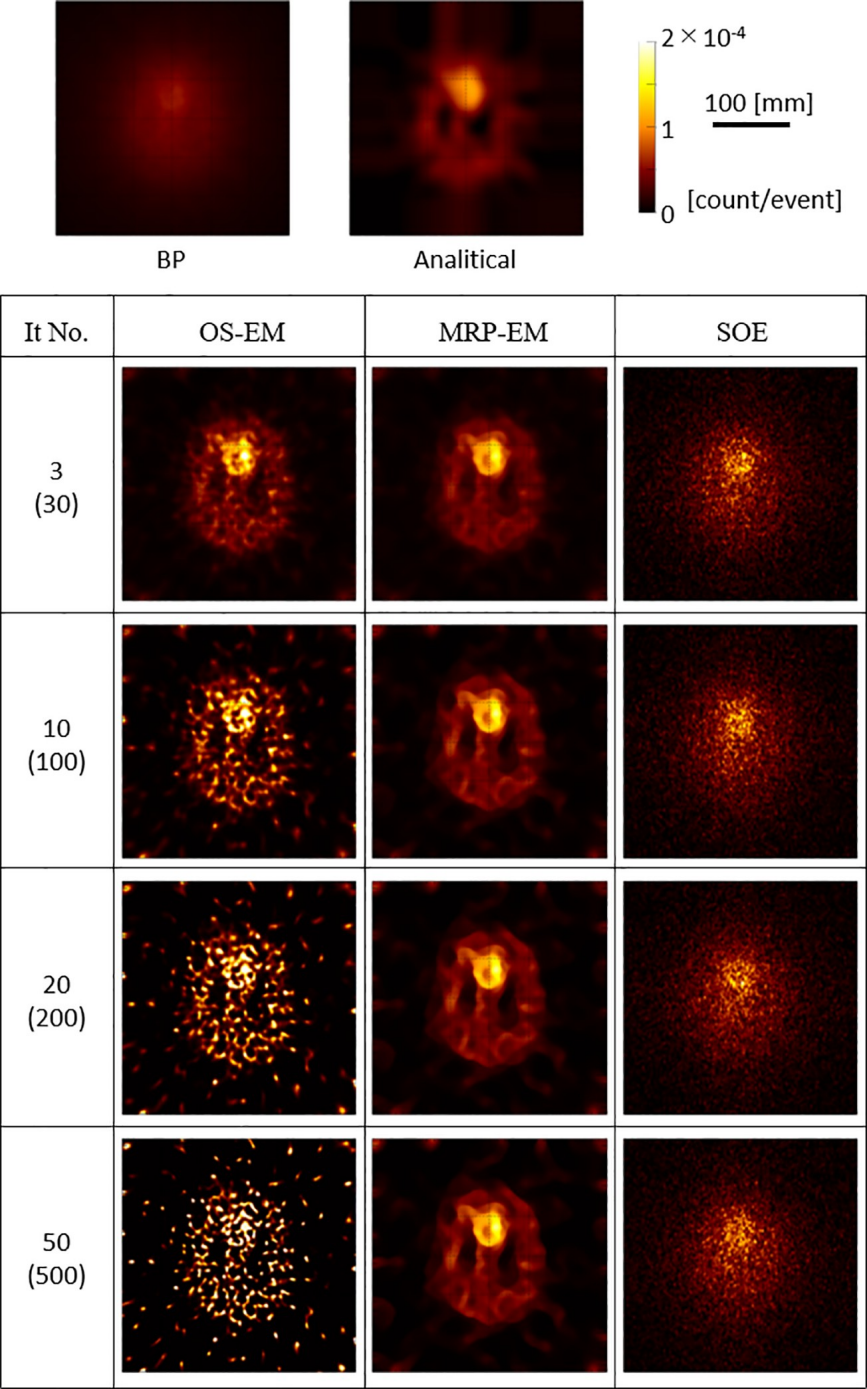
Images reconstructed using the various algorithms. Numbers in brackets denote the number of iterations for SOE (10 times more than for OS-EM and MRP-EM).

The Monte Carlo simulation performed on the ellipsoidal phantom (Fig 1(B)) measured 23648 Compton events. Fig 4 shows the reconstructed images of the phantom using 3, 10, 20, and 50 iterations of the OS-EM and MRP-EM algorithms and 30, 100, 200, and 500 iterations (10 times more than OS-EM and MRP-EM) of the SOE algorithm, along with the images reconstructed by the BP and Analytic algorithms.

**Fig 5:**
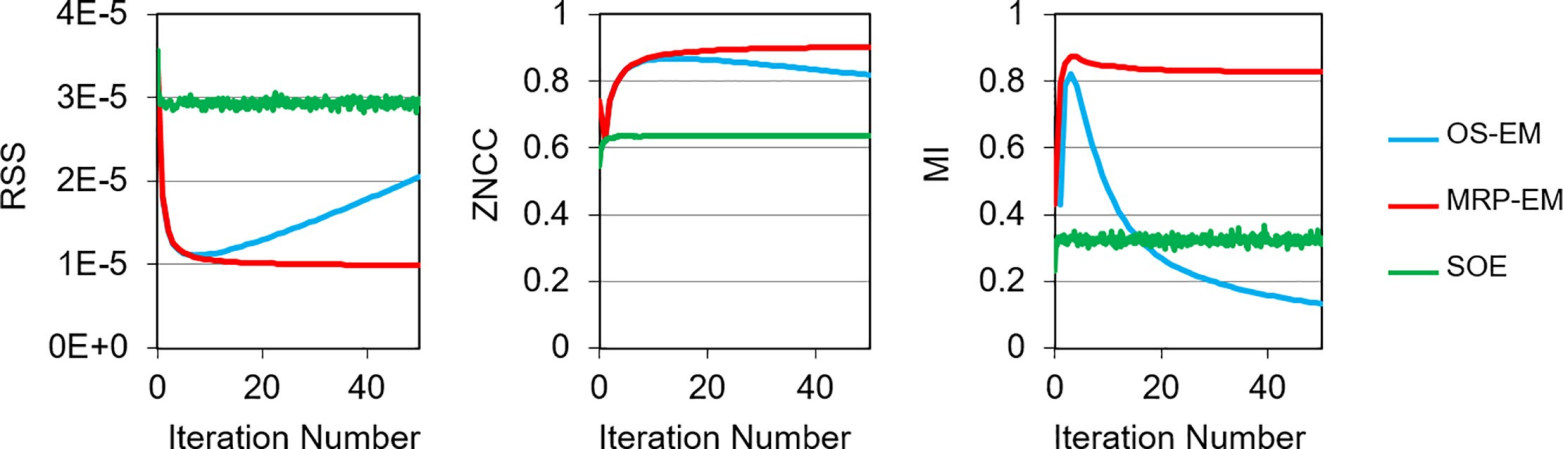
Changes in RSS, ZNCC, and MI of the reconstructed image for each iterative algorithm (OS-EM: blue, MRP-EM: red and SOE: green). Lower values are preferable for RSS, and higher values are better for ZNCC and MI. The x-axis value (iteration number) of SOE is 10 times greater than that of OS-EM and MRP-EM. The RSS, ZNCC, and MI values of BP were 2.8×10^−5^, 0.35, and 0.43, respectively, and those of the analytical method were 2.0×10^−5^, 0.88, and 0.76, respectively.

The change in RSS, ZNCC, and MI with respect to the number of iterations is shown for each iterative algorithm (OS-EM, MRP-EM, and SOE) in Fig 5. In the case of OS-EM, the image quality initially increases, but decreases as the number of iterations becomes excessive. The optimal number of iterations is different for each index (RSS, ZNCC, and MI). The image quality of MRP-EM increases monotonically for RSS and ZNCC, but, after an initial increase, decreases to a constant value in the case of MI. The image quality of SOE remains almost constant in each case. The RSS, ZNCC, and MI values of BP were 2.8×10^−5^, 0.35, and 0.43, respectively, and those of the analytical method were 2.0×10^−5^, 0.88, and 0.76, respectively.

**Fig 6:**
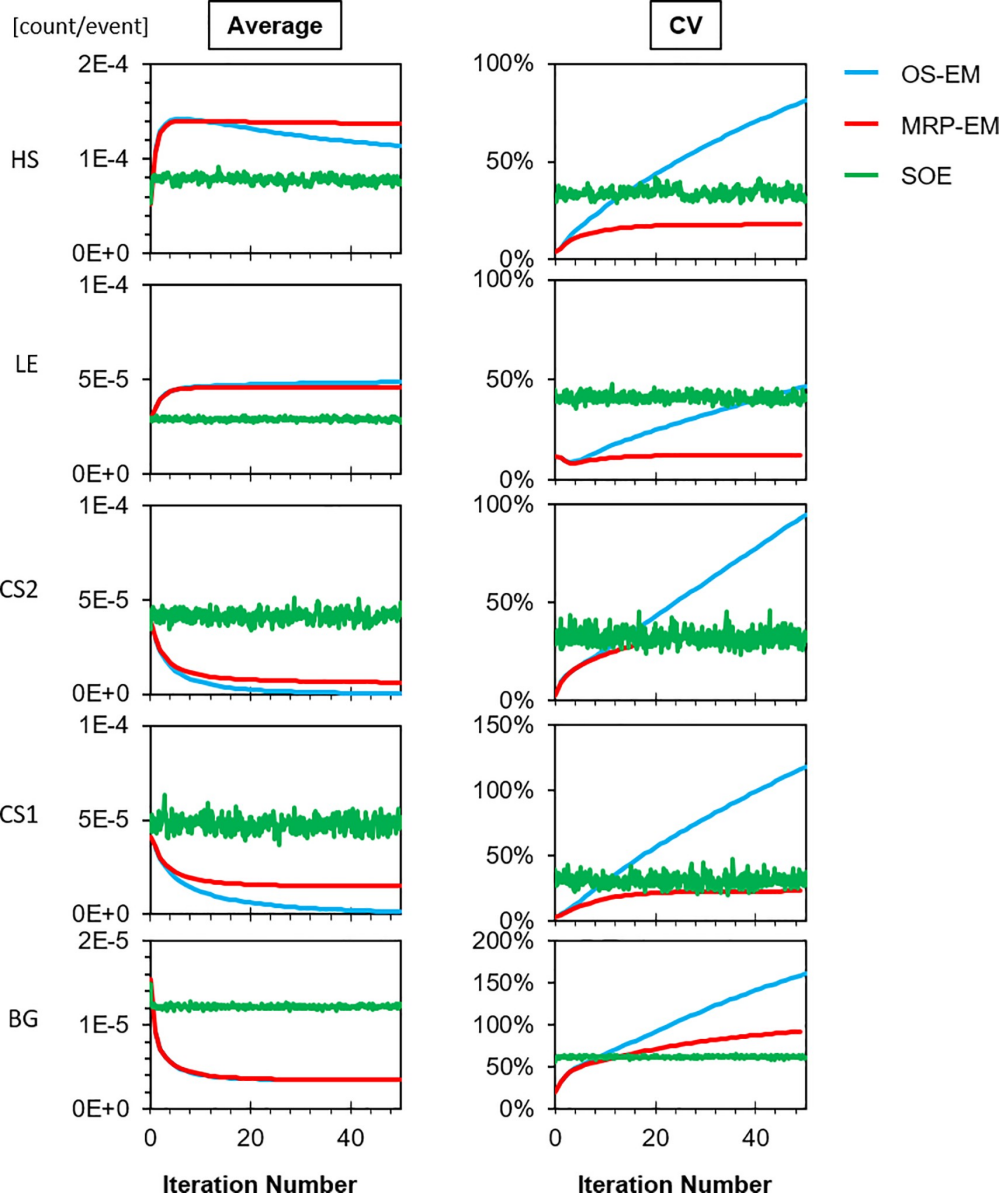
Changes in average and CV of HS, LE, CS1, CS2, and BG of the reconstructed images for each iterative algorithm (OS-EM: blue, MRP-EM: red and SOE: green). The x-axis value (iteration number) of SOE is 10 times greater than that of OS-EM and MRP-EM. The average values (CV) of HS, LE, CS1, CS2, and BG for the analytical method were 1.0×10^−4^ (6.8%), 4.1×10^−5^ (10%), 1.6×10^−5^ (36%), 1.0×10^−5^ (73%), and 9.3×10^−6^ (65%), and those for BP were 6.8×10^−5^ (3.6%), 3.0×10^−5^ (12%), 4.1×10^−5^ (3.0%), 3.7×10^−5^ (4.8%), and 1.3×10^−5^ (23%).

The change in the average and CV of the reconstructed image for each iterative algorithm is shown in Fig 6. The true values in HS and LE were 1.4×10^−4^ and 4.8×10^−5^, respectively. The average values (CV) of HS, LE, CS1, CS2, and BG for the analytical method were 1.0×10^−4^ (6.8%), 4.1×10^−5^ (10%), 1.6×10^−5^ (36%), 1.0×10^−5^ (73%), and 9.3×10^−6^ (65%), respectively, and those for BP were 6.8×10^−5^ (3.6%), 3.0×10^−5^ (12%), 4.1×10^−5^ (3.0%), 3.7×10^−5^ (4.8%), and 1.3×10^−5^ (23%), respectively.

Using MRP-EM, the average values of all regions asymptotically approached a stable value, and the CV also became stable; this is in contrast to OS-EM, where the average and CV continued to change on each iteration. Both the average and CV values remained almost unchanged in the case of SOE.

**Fig 7:**
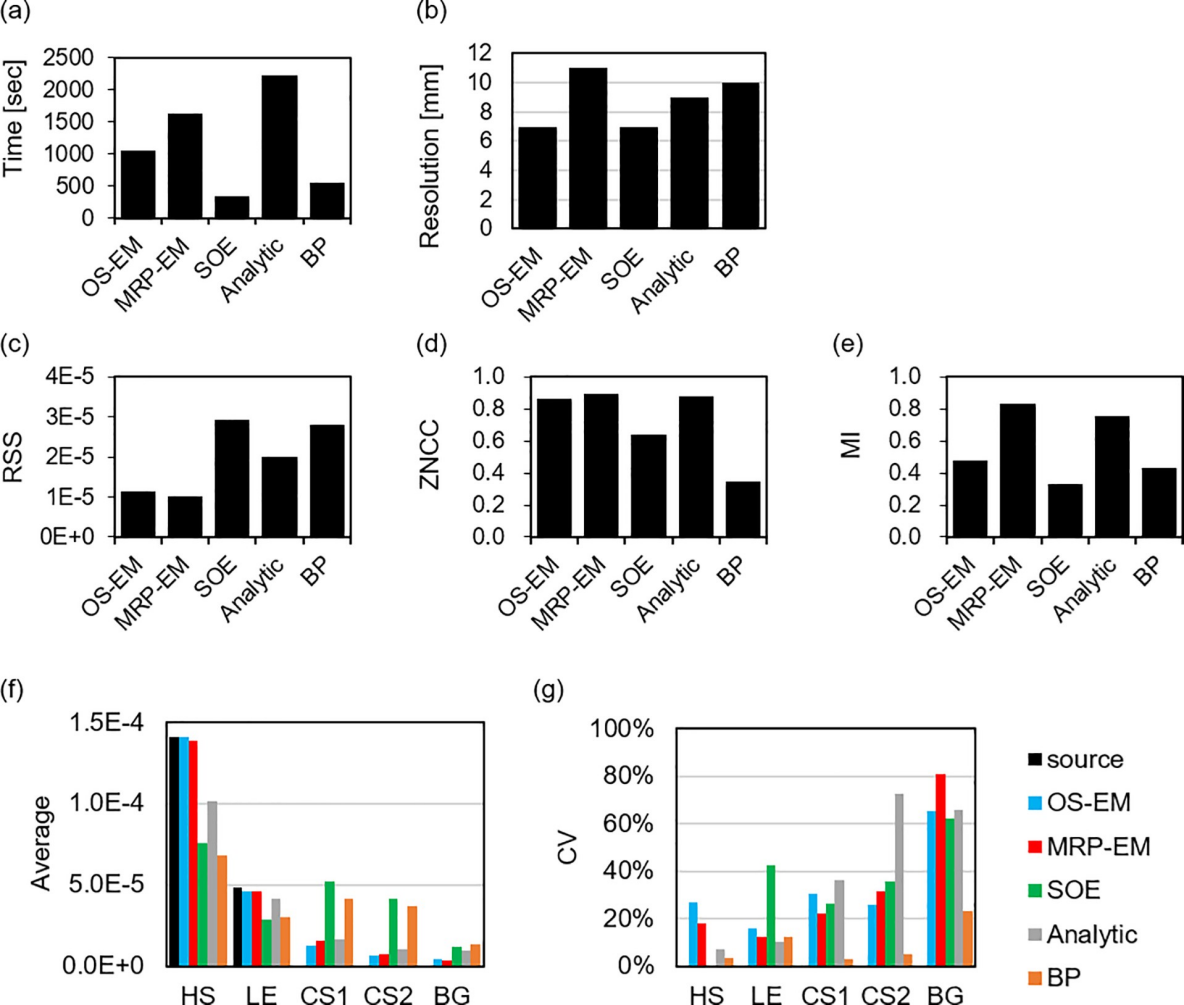
Comparison of image quality of BP, Analytic algorithm, OS-EM with 10 iterations, MRP-EM with 20 iterations, and SOE with 200 iterations. (a) Reconstruction time (shorter is better), (b) spatial resolution (shorter is better), (c) RSS (smaller is better), (d) ZNCC (higher is better), (e) MI (higher is better), (f) average values in each position, and (g) CV in each position.

Finally, the image quality after a reasonable number of iterations is compared considering RSS, ZNCC, and MI values, in Fig 7. From Fig 5, the optimal numbers of iterations for OS-EM, MRP-EM, and SOE were set to 10, 20, and 200, respectively.

## 4. Discussion

We evaluated the imaging properties of the MRP-EM algorithm against previous methods. We adopted SOE as an iterative algorithm without ML-EM (and advanced method of ML-EM), as well as the analytical method developed by Tomitani and Hirasawa. These methods have been used in previous comparison studies [28–31]. Nuclear medical images require the ability to identify disease. Unfortunately, there is no versatile index that can measure the degree of “identifiability” in nuclear medicine diagnosis. In this study, to evaluate the difference in the reconstructed images with respect to the original source distribution, we used the RSS, ZNCC, and MI metrics. There is a possibility that images with similar index values may exhibit quite different qualities [32]. RSS evaluates the difference directly, ZNCC estimates the difference by reducing the effect of the background, and MI is insensitive to impulsive noise [33]. In addition, the images reconstructed with a reasonable number of iterations were compared to evaluate the reconstruction ability on a fair basis (Fig 7).

When OS-EM was used to reconstruct images of the point sources, the pixel values were excessively concentrated in one spot. Thus, the spatial resolution could not be evaluated using a point spread function. Therefore, two point sources placed at various distances apart (5–15 mm) were imaged to evaluate the smallest distance between two distinguishable points, defined as the spatial resolution. It was found that OS-EM and SOE possesses the best spatial resolution, although that of the proposed MRP-EM was only slightly worse. It is not surprising that a smoothing filter degrades the spatial resolution of images.

Fig 4 shows the reconstructed images of the ellipsoidal phantom. As shown in Fig 7, OS-EM can reconstruct an image with good spatial resolution in a short time, if the number of iterations is appropriate. However, the image quality drastically changes with the number of iterations (Fig 5). With ML-EM, the image is updated iteratively, and OS-EM accelerated the process. Thus, it can be concluded that the image reconstructed by OS-EM is very sensitive to the iteration number. The optimal number of iterations depends on the data size and the complexity of the source distribution. Hence, it is difficult to optimize the number of iterations in a general way. The use of a median filter can reduce noise and facilitate the adoption of the best iteration number. In our preliminary experiment, the median filter was found to prevent the excess accumulation more efficiently than a mean filter and Gaussian filter (data not shown). The convergence time for MRP-EM is longer than that of OS-EM. This is mainly due to the number of required iterations for convergence instead of the increase in calculation time for an iteration. We believe the computation time is acceptable. In addition, MRP-EM produced better values of RSS, ZNCC, and MI than the other reconstruction methods (including OS-EM). This means that MRP-EM provides better images than OS-EM, if the unacceptable noise could be avoided by an appropriate early termination method. OS-EM statistically estimates the source distribution, resulting in statistical noises in the image being emphasized easily. In general, medical images are locally monotonic. Thus, the median filter smoothens images, and MRP-EM can achieve feasible images by avoiding impulse noises.

The stability of MRP-EM against the number of iterations was also confirmed through semi-quantitative and uniformity analysis (Fig 6). In the case of OS-EM, the CV value increased monotonically and exhibited the tendency of OS-EM to over-concentrate the image in one spot. The average value of HS with OS-EM displayed a peak and departed from the true value as the iterations continued, resulting in the curve shown in Fig 4. The average values in CS1 and CS2 under the OS-EM approach tend asymptotically to zero, but the CV values increased rapidly and the cold spots were unclear in the reconstructed images. Conversely, the RSS and ZNCC values with MRP-EM improved with the number of iterations. Though the MI value with MRP-EM decreased after a peak value, the curve became stable near the peak. The average values in CS1 and CS2 with MRP-EM remained constant. However, the cold spots can be identified in the reconstructed image. The average values of CS1 and CS2 depend on the size of the cold spot.

In this study, SOE was found to reconstruct the image very quickly, even though we used more iterations in accordance with previous reports [15, 34]. However, the image quality was not good with respect to the measured values of RSS, ZNCC, and MI. Many pixel values remained in the cold spots or BG region. This was because the number of Compton events used for the reconstruction was too small compared to the number of pixels (90000), and sufficient possible origin-points could not be accumulated in the hot spot. It is obvious that image reconstruction with a small number of pixels produces a better image (data not shown). The advantage of SOE is its speedy reconstruction, even with a large number of Compton events. Unfortunately, this merit was not examined with a relatively small number of Compton events in this study.

The analytical method developed by Tomitani and Hirasawa can reconstruct good images while maintaining the spatial resolution and has low computer memory requirements. Comparing MRP-EM and the analytical method, the computation time, ZNCC, and MI results were comparable, but MRP-EM achieved a better RSS.

The proposed MRP-EM requires considerable computational resources. In particular, the calculation time and memory requirements become significant as the number of Compton events increases. In a human experiment [35], it was found that Compton images could be reconstructed using fewer Compton events. Thus, the conditions of this study were not unrealistic. With improvements in Compton cameras, the number of Compton events available could be increased. On the other hand, the pixel size could be larger considering the spatial resolution of Compton camera. This aspect requires further investigation. At least, the parameter *β* of MRP-EM and the number of subsets have to be optimized in tune with the imaging conditions. A larger number of subsets accelerates the convergence rate, but the data size of each subset contains less statistical information. This would result in enhanced noise structures in the final image [36]. β is an intensity factor of the filter of MRP-EM. Small numerical values of β cannot reduce the noise, and the image would be similar to the image obtained with OS-EM. In contrast, some reduction in the spatial resolution was observed in this study with β = 1, which is a drawback of MRP-EM. A right combination of β and filter size could improve image quality without a considerable resolution reduction. Further research is required to investigate appropriate values.

MRP-EM can be easily extended to three dimensions. Additionally, the use of GPUs would speed up the reconstruction time, because MRP-EM is parallelizable. Thus, we believe that MRP-EM is a promising algorithm for nuclear medical applications.

## 5. Conclusions

The main goal of this study was to evaluate the ability of MRP-EM, which is a modified version of OS-EM, with a median filter. MRP-EM can produce high-quality reconstructed images without over-concentration in a reasonable computation time. Though the spatial resolution is slightly worse than that of OS-EM, the image quality indices evaluated in this study suggest that MRP-EM provides better reconstructions than other analytical or iterative methods. We will extend the MRP-EM algorithm to three dimensions as the next step.

## Supporting information

S1 Fig Compton images of simulation study reconstructed by ML-EM algorithm with respect to iteration number (3, 10, 20, and 50) and the size of the median mask (3×3, 5×5, 7×7, and 11×11). (PDF)

S2 Fig RSS and ZNCC evaluation for different sizes of median mask (3×3, 5×5, 7×7, and 11×11) of the simulated Compton images reconstructed by ML-EM algorithm. Lower values are preferable for RSS, and higher values are better for ZNCC and MI.
(PDF)
