# Supplementary material for: Improved iterative reconstruction method for Compton imaging using median filter
Source: PLoS One. 2020 Mar 6;15(3):e0229366. doi: 10.1371/journal.pone.0229366 (PMC7059936; doi:10.1371/journal.pone.0229366)
Supplement: S1 Fig — (PDF) [file pone.0229366.s001.pdf]

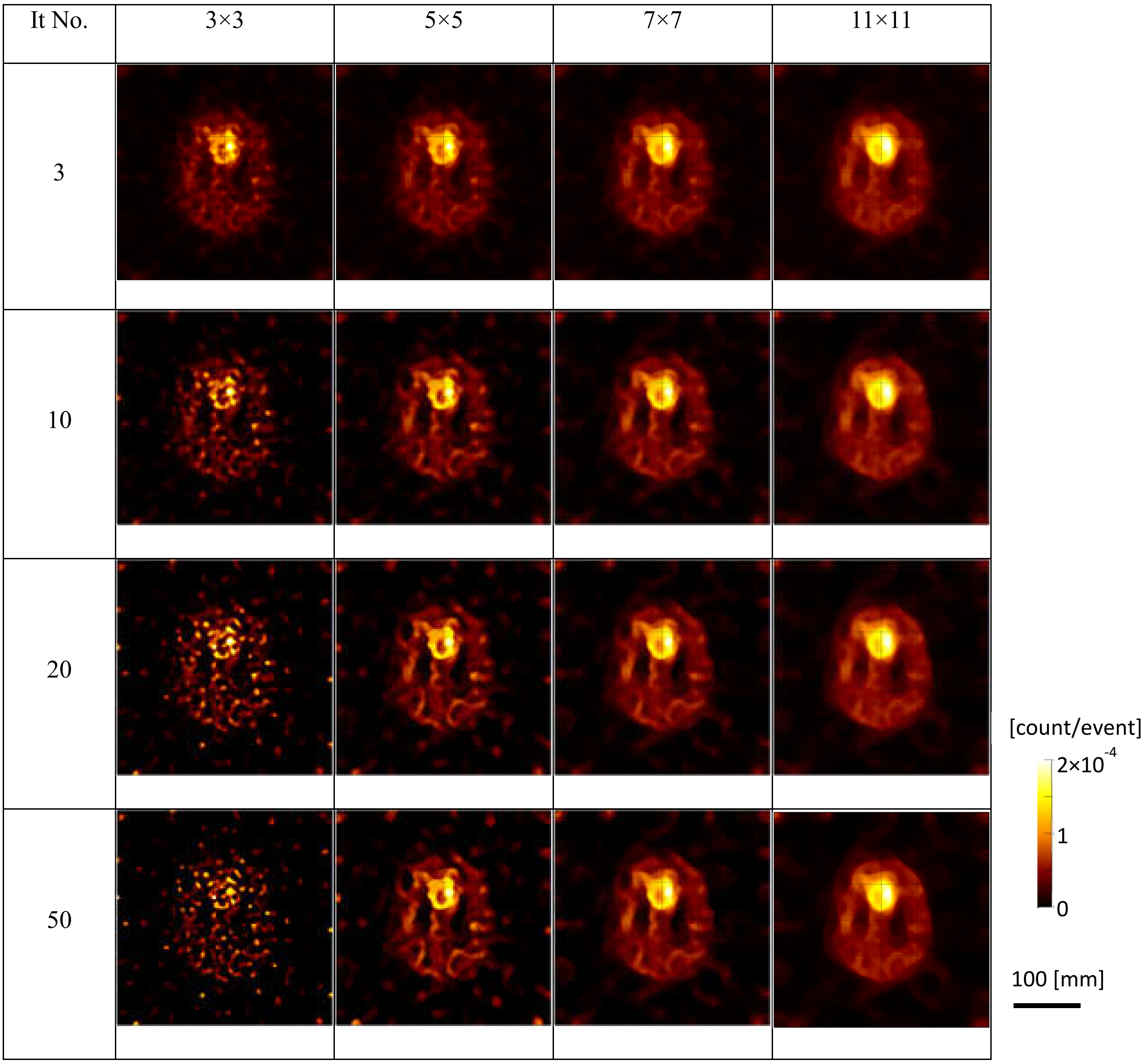

Supporting Figure 1. Compton images of simulation study reconstructed by ML-EM algorithm with respect to iteration number (3, 10, 20, and 50) and the size of the median mask (3×3, 5×5, 7×7, and 11×11).
