# Supplementary material for: Improved iterative reconstruction method for Compton imaging using median filter
Source: PLoS One. 2020 Mar 6;15(3):e0229366. doi: 10.1371/journal.pone.0229366 (PMC7059936; doi:10.1371/journal.pone.0229366)
Supplement: S2 Fig — Lower values are preferable for RSS, and higher values are better for ZNCC and MI. (PDF) [file pone.0229366.s002.pdf]

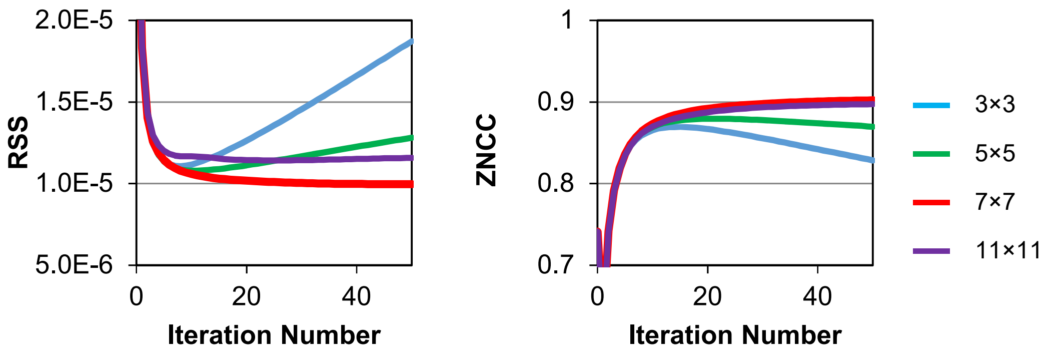

RSS ZNCC

Supporting Figure 2. RSS and ZNCC evaluation for different sizes of median mask (3×3, 5×5, 7×7, and 11×11) of the simulated Compton images reconstructed by ML-EM algorithm. Lower values are preferable for RSS, and higher values are better for ZNCC and MI.
